# Evidence that two instead of one defective interfering RNA in influenza A virus-derived defective interfering particles (DIPs) does not enhance antiviral activity

**DOI:** 10.1038/s41598-021-99691-1

**Published:** 2021-10-14

**Authors:** Najat Bdeir, Prerna Arora, Sabine Gärtner, Stefan Pöhlmann, Michael Winkler

**Affiliations:** 1 Infection Biology Unit, German Primate Center, Göttingen, Germany; 2 Faculty of Biology and Psychology, University Göttingen, Göttingen, Germany

**Keywords:** Virology, Influenza virus

## Abstract

Influenza A virus (IAV) infection constitutes a significant health threat. Defective interfering particles (DIPs) can arise during IAV infection and inhibit spread of wild type (WT) IAV. DIPs harbor defective RNA segments, termed DI RNAs, that usually contain internal deletions and interfere with replication of WT viral RNA segments. Here, we asked whether DIPs harboring two instead of one DI RNA exert increased antiviral activity. For this, we focused on DI RNAs derived from segments 1 and 3, which encode the polymerase subunits PB2 and PA, respectively. We demonstrate the successful production of DIPs harboring deletions in segments 1 and/or 3, using cell lines that co-express PB2 and PA. Further, we demonstrate that DIPs harboring two instead of one DI RNA do not exhibit increased ability to inhibit replication of a WT RNA segment. Similarly, the presence of two DI RNAs did not augment the induction of the interferon-stimulated gene MxA and the inhibition of IAV infection. Collectively, our findings suggest that the presence of multiple DI RNAs derived from genomic segments encoding polymerase subunits might not result in increased antiviral activity.

## Introduction

Influenza A viruses (IAVs) are a global health threat responsible for annual epidemics and occasional pandemics^1,2^. Currently available influenza therapy includes M2 ion channel inhibitors (Rimantadine and Amantadine)^3^, neuraminidase inhibitors (Zanamivir and Oseltamivir)^4,5^, and an inhibitor of the viral polymerase (Baloxavir marboxil)^4–6^. However, resistance mutations can render these drugs ineffective^7^. Similarly, vaccines against epidemic influenza have to be reformulated on an annual basis due to antigenic drift of the circulating influenza virus strains and offer little or no protection against newly emerging, pandemic strains^8,9^. Hence, there is an urgent need for the development of novel prophylactic and therapeutic strategies.

Influenza viruses are enveloped and harbor a negative-sense, segmented RNA genome. The viral nucleoprotein (NP) and the trimeric viral polymerase consisting of the subunits polymerase basic 1 (PB1), polymerase basic 2 (PB2) and polymerase acidic (PA) are required for genome replication^9,10^. Errors made by the viral polymerase during genome replication may result in the production of defective genomic RNAs, which frequently harbor deletions^11^. Some of these defective RNAs interfere with replication of WT RNAs and the packaging of these defective interfering (DI) RNAs into particles yields DI particles, DIPs^11,12^. DIPs inhibit infection with WT influenza viruses by interfering with genome replication and by inducing the expression of interferon stimulated genes (ISGs), including the MxA gene. DIPs can modulate influenza virus spread in the host and could be developed for antiviral therapy^13,14^. However, it is at present unclear whether DIPs harboring more than one DI RNA will exert increased antiviral activity as compared to otherwise isogenic DIPs harboring a single DI RNA.

We have reported the establishment of a cell culture system which allows the production of DIPs bearing a segment 1-derived DI RNA in the absence of infectious virus^15^. Here, we modified this system in order to produce DIPs harboring DI RNAs derived from segments 1 and 3. We found that DIPs harboring DI RNAs derived from segments 1 and 3 can be readily generated in this system but do not show augmented antiviral activity as compared to DIPs harboring a single DI RNA.

## Results

### Generation of 293T and MDCK cells stably expressing functional PA and PB2

**Figure 1:**
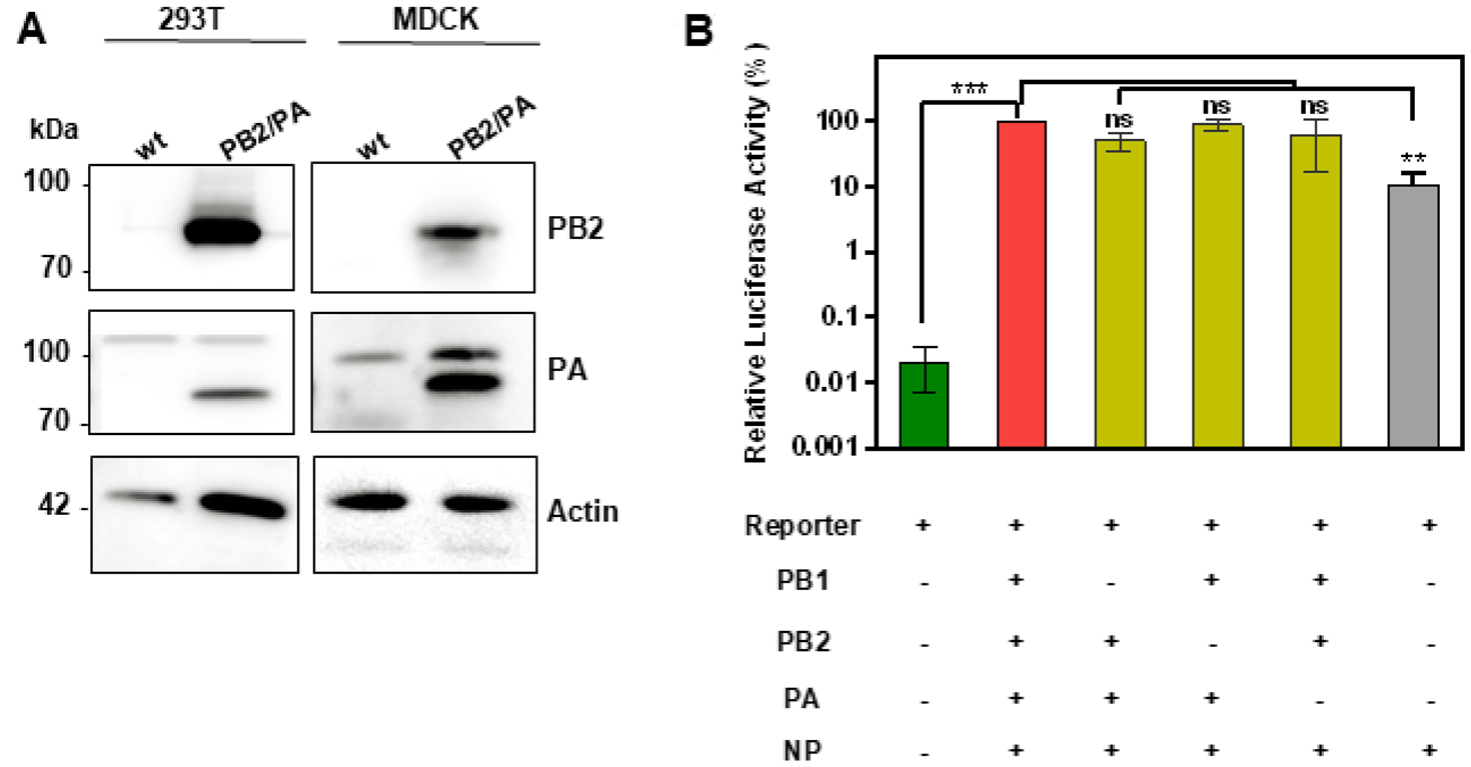
Characterization of MDCK and 293 T cells stably expressing IAV polymerase proteins. (**A**) Expression of PB2 and PA in MDCK and 293 T cells was analyzed by immunoblot. Detection of β-actin served as a loading control. Lysates from MDCK WT/MDCK PB2/PA and 293 T WT/293 T PB1/PB2/PA cells were loaded on three gels. Blots were exposed for 50 s for detection of PB2 in 293 T cells and for 30 s for detection in MDCK cells while exposure of 50 s was required for detection of β-actin. The exposure for detection of PA in 293 T and MDCK cells was 50 s and 10 s, respectively. (**B**) 293 T cells stably expressing PB2, PB1, and PA were transiently co-transfected with the indicated combinations of plasmids encoding PB2, PB1, PA and NP and an IAV luciferase reporter segment. Luciferase activities in cell lysates were determined at 24 h post transfection. Luciferase activity measured for cells co-transfected with all plasmids was set as 100%. The average of three independent experiments is shown. Error bars indicate SEM. Two tailed paired student t-test was used to assess statistical significance. p < 0.05 = **, p < 0.005 = ***, ns: not significant.

In order to determine whether DIPs harboring more than one DI RNA exert increased antiviral activity, we focused on segment 1- and segment 3-derived DI RNAs, since DI RNAs most frequently arise from genomic segments 1–3, which encode polymerase proteins^16,17^. We have previously generated a cell line that stably expresses PB2 (which is encoded by segment 1) and allows amplification of DIPs harboring segment 1-derived DI RNAs^15^. For production of DIPs harboring segment 1- and/or 3-derived DI RNAs we employed the same strategy. Thus, we engineered 293 T and MDCK cells to co-express PB2 and PA (encoded by segment 3). Immunoblot analysis revealed that these cells indeed expressed robust levels of the desired proteins (Fig. 1A and supplemental Fig. 1). In addition, the 293T cell line was engineered to express PB1 and expression was readily detectable (Supplemental Fig. 1). In order to analyze whether the PB2, PB1 and PA stably expressed in 293T cells are functional, we used a mini replicon system. This assay measures amplification of a reporter segment (derived from segment 8) encoding luciferase upon co-expression of PB1, PB2, PA and NP^18^. We found that transfection of this 293T cell line with a plasmid encoding the IAV reporter segment yielded background levels of luciferase activity, while co-transfection of these cells with plasmids encoding the reporter segment and PB2, PB1, PA and NP increased luciferase activity more than 1000-fold (Fig. 1B). Notably, the omission of plasmids encoding PB2, PB1 and PA did not appreciably reduce reporter activity (Fig. 1B); demonstrating that our 293T cell line expressed functional PB2, PB1 and PA. Comparable functionality could not be analyzed for the MDCK cell line expressing PA and PB2 due to low transfection efficiency (not shown).

### PB2 and PA co-expressing cell lines allow production of DIPs harboring segment 1- and/or segment 3-derived DI RNAs

**Figure 2:**
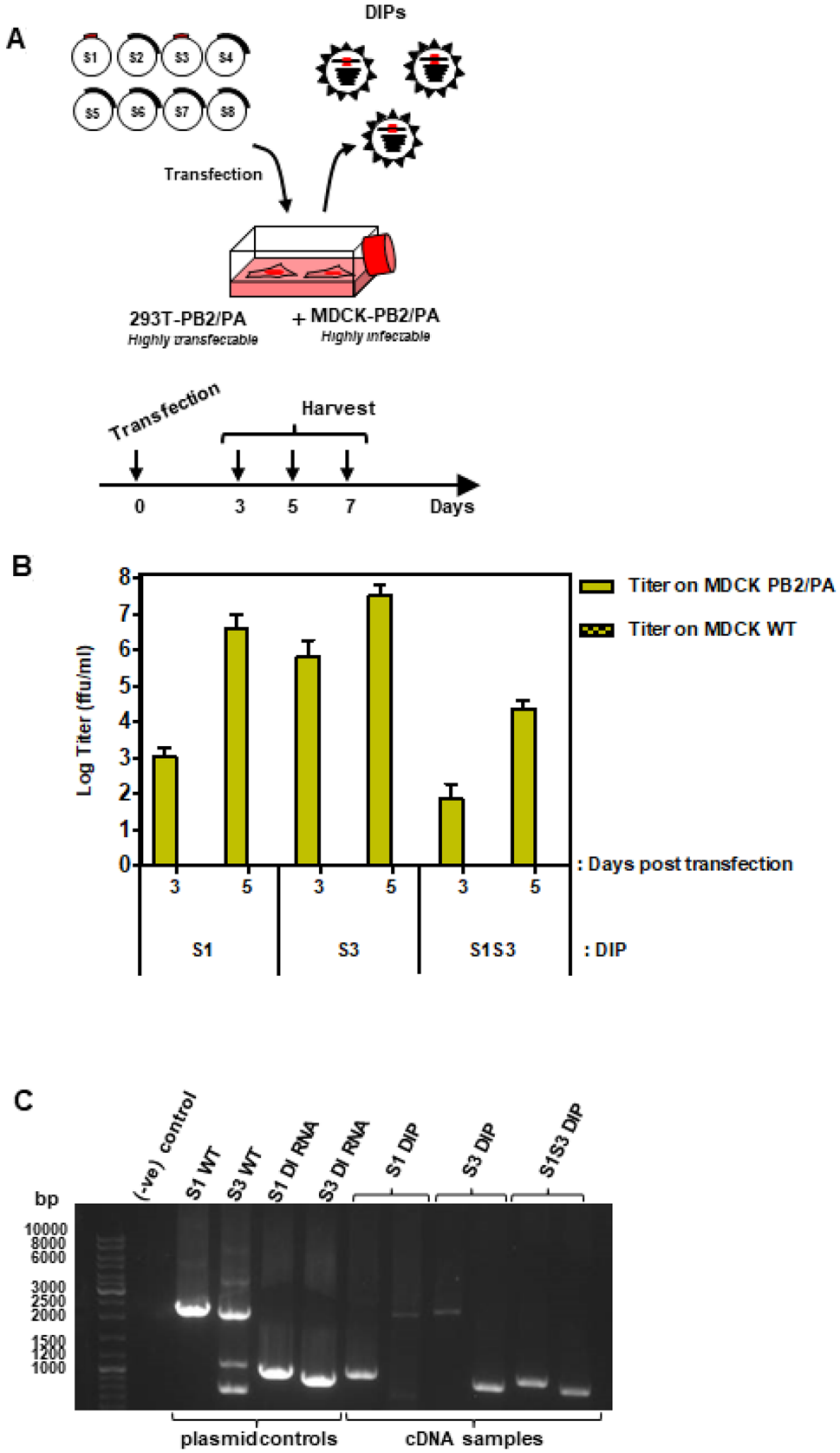
Production of DIPs from complementing cell lines. (**A**) Work flow for production and quantification of DIPs harboring segment 1- and 3-derived DI RNAs. For production of DIPs, a co-culture of 293T and MDCK cells stably expressing at least two IAV polymerase proteins was co-transfected with plasmids encoding WT IAV genomic segments 2 and 4–8 as well as segments 1 and 3 containing internal deletions that convert these segments into DI RNAs. Supernatants were harvested at the indicated time points. (Modified from^15^, URL: https://journals.plos.org/plosone/article?id=10.1371/jornal.pone.0212757, under the creative commons attributions license link: https://creativecommons.org/licenses/bi/4.0/) (**B**) A co-culture of PB1/PB2/PA 293T cells and PB2/PA MDCK cells was transfected with plasmids encoding S1, S3 or S1S3 DI RNAs jointly with the remaining IAV WT genomic segments. Supernatants were harvested on days 3, 5 and 7-post transfection. Titers were determined by focus formation assay using PA/PB2 MDCK cells. The average of three independent experiments is shown. Error bars indicate SEM. (**C**) DI RNA incorporation into DIPs. Supernatants containing DIPs harboring DI RNAs derived from segment 1 (S1) or 3 (S3) or harboring both DI RNAs (S1S3) were analyzed by RT-PCR. Plasmids encoding wt segments S1, S3 or the corresponding DI RNAs served as positive control. A single representative experiment is shown. Results were confirmed in four independent experiments.

We next explored whether the generated stable cell lines allowed production of DIPs harboring a segment 1-derived DI RNA (S1 DIP), a segment 3-derived DI RNA (S3 DIP) or a segment 1- and a segment 3-derived DI RNA (S1S3 DIP), employing the experimental setup depicted in Fig. 2A. For DIP production, segment 1- and 3-derived derived DI RNAs with an internal deletion of 1248 nts (S1) and 1193 nts (S3) were chosen based on our unpublished findings indicating that these DI RNAs induce replication interference and are compatible with robust DIP production. Quantification of DIP infectivity by focus formation assay revealed titers of roughly 10^7^ ffu/mL for S1 and S3 DIPs and 10^4^ ffu/mL for S1S3 DIPs (Fig. 2B). In contrast, no DIPs were produced when 293T WT and MDCK WT cells were used for production (Fig. 2B). Next, we sought to confirm the incorporation of S1 and S3 DI RNAs into DIPs via segment specific RT-PCR. The RT-PCR yielded bands of the expected sizes, 1032 bp for S1 DI RNA, 2.28 kbp for WT segment 1, 958 bp for S3 DI RNA and 2.151 kbp for segment 3 WT. Importantly, S1 DI RNA but not WT segment 1 was detected in S1 DIPs and the corresponding observation was made for WT segment 3 and S3 DIPs (Fig. 2C). Similarly, S1 DI RNA and S3 DI RNA but not the corresponding WT segments were detected in S1S3 DIPs, confirming the purity of our DIP preparations (Fig. 2C). Thus, the newly established cell lines allowed production of S1, S3 and S1S3 DIPs harboring the desired DI RNAs.

### Co-expression of S1 and S3 DI RNAs does not augment inhibition of segment replication

**Figure 3:**
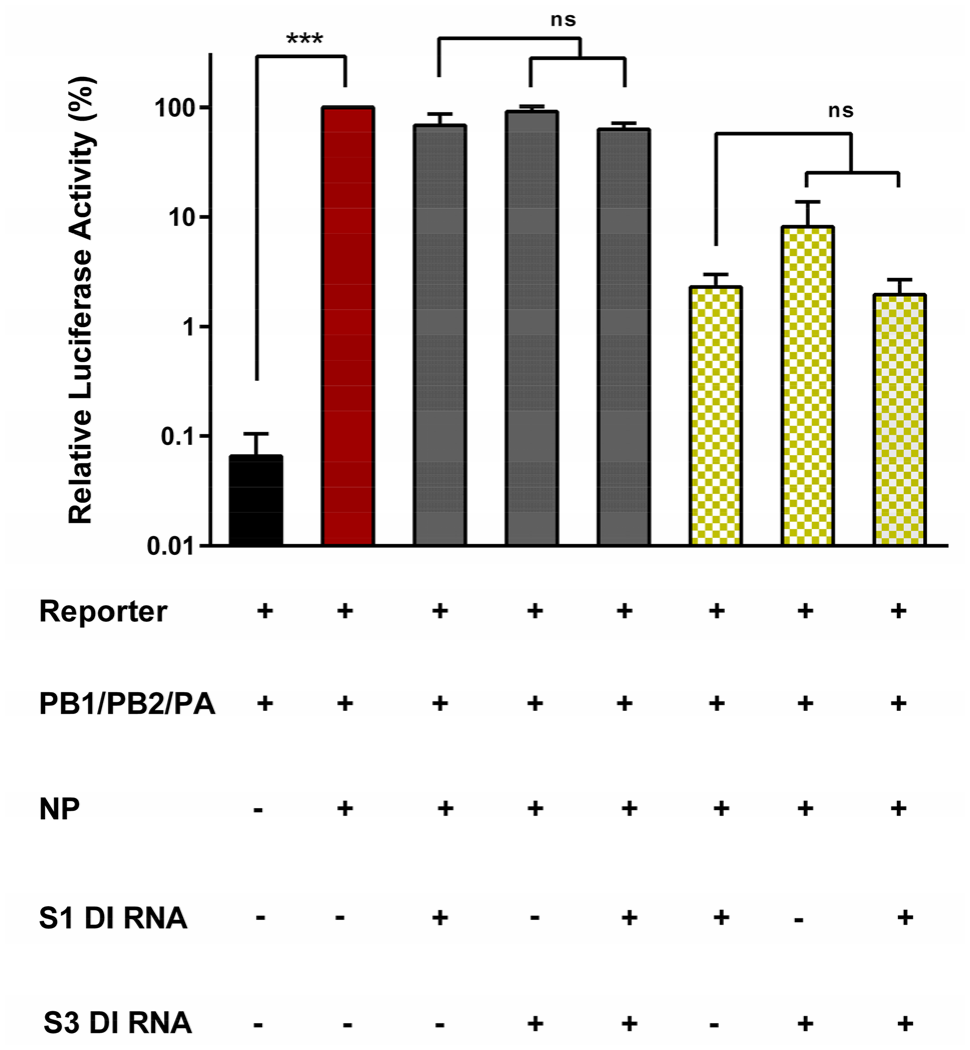
The presence of two DI RNAs does not increase inhibition of genome replication. 293T cells were cotransfected with the indicated combinations of plasmids encoding the viral polymerase proteins, NP, a segment 8-based luciferase reporter and the indicated DI RNAs at 10 (gray bars) and 300 ng (green checkered bars). Luciferase activity was determined at 24 h posttransfection. The results of single round DI RNA induced replication inhibition are shown. The average of three independent experiments is shown. Error bars indicate SEM. Two tailed paired student t-test was used to assess statistical significance. p < 0.005 = ***, ns: not significant.

DI RNAs suppress replication of WT IAV genomic segments and we investigated whether combining S1 DI RNA and S3 DI RNA increases inhibitory activity as compared to the single DI RNAs. For this purpose, we employed the mini replicon assay as described above but used 293T WT cells. Transfection of 293T cells with the IAV reporter segment alone yielded background levels of luciferase activity, while cotransfection of these cells with plasmids encoding the reporter, IAV polymerase proteins and NP yielded luciferase levels 1000-fold over background. Additional cotransfection of plasmids encoding S1 DI RNA or S3 DI RNA at two concentrations resulted in a dose-dependent decrease of luciferase activity, with S1 DI RNA showing a stronger inhibitory effect as compared to S3 DI RNA (Fig. 3). Further, cotransfection of plasmids encoding both DI RNAs did not result in further decrease of luciferase activity as compared to that measured for S1 DI RNA alone (Fig. 3), indicating that the presence of two DI RNAs did not increase replication interference in the mini replicon assay.

### S1S3 DIPs do not induce MxA expression with increased efficiency as compared to S1 or S3 DIPs

**Figure 4:**
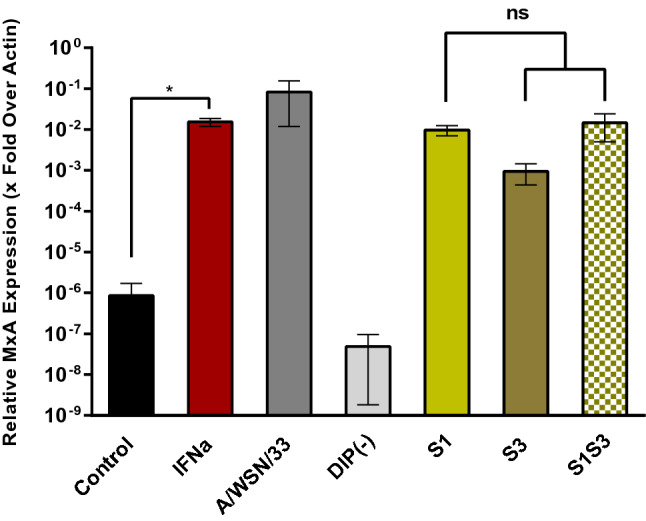
The presence of two DI RNAs does not increase MxA induction. Calu-3 cells were either treated with interferon α or inoculated with IAV (A/WSN/33) or DIPs at an MOI of 0.1. Cell lysates were harvested 24 h after treatment or infection, RNA was isolated and MxA expression was analyzed by quantitative real time PCR. MxA transcript levels were normalized against transcript levels of β-actin. The average of three independent experiments is shown as x-fold over actin. Error bars indicate SEM. Two tailed paired student t-test was used to assess statistical significance. p < 0.5 = *, ns: not significant.

DIPs can inhibit heterologous viruses by inducing the IFN system^19^. Therefore, we assessed whether the presence of two DI RNAs as compared to one effected a stronger induction of MxA expression, an IFN stimulated gene. Interferon α or infection with A/WSN/33 induced MxA expression at least 100-fold, as determined by quantitative RT-PCR (Fig. 4). S1 and S3 DIPs induced MxA with similar efficiency as A/WSN/33, but MxA induction was not augmented when S1S3 DIPs harboring two DI RNAs were studied (Fig. 4). Thus, combining S1 and S3 DI RNA in DIPs did not enhance induction of MxA expression.

### S1S3 DIPs do not show increased antiviral activity as compared to S1 or S3 DIPs

We finally investigated whether the presence of two DI RNAs increases DIP antiviral activity, i.e. the ability to suppress replication of WT IAV in target cells. For this, we used MDCK cells, a dog cell line frequently used to propagate IAV. Furthermore, we employed the human lung cell line Calu-3 as a mimic of human respiratory epithelium. In order to assess DIP antiviral activity in these cell lines, different DIP dilutions were either added 24 h prior to virus (24 h setting) or DIP and virus were added at the same time (0 h setting).

**Figure 5:**
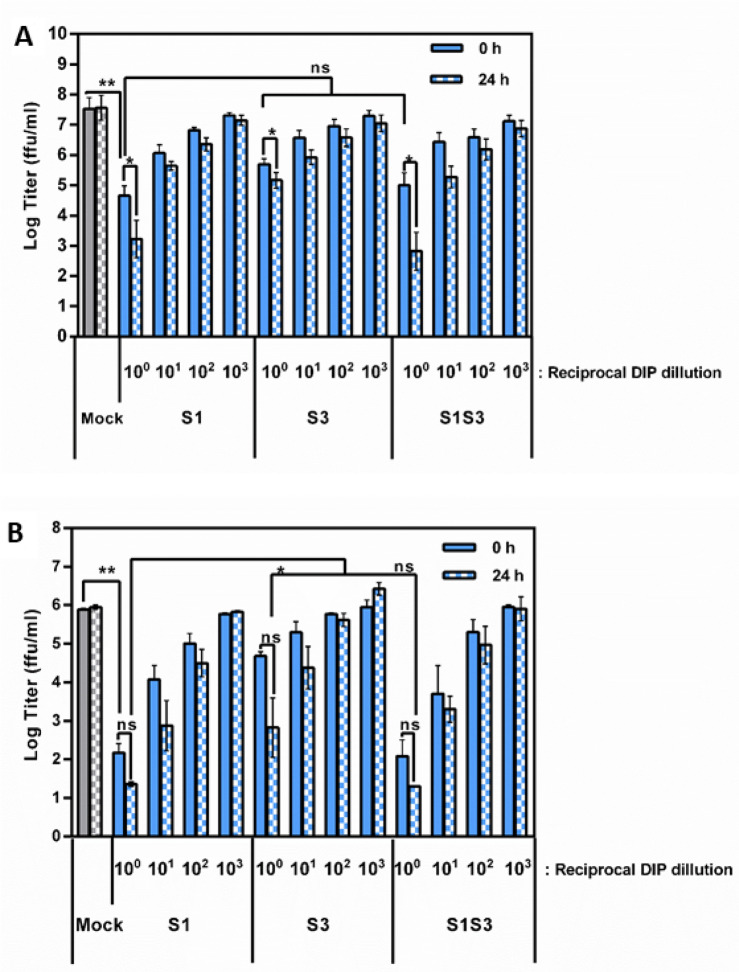
The presence of two DI RNAs does not increase antiviral activity. (**A**) MDCK cells were either co-inoculated with A/WSN/33 and the indicated DIPs at the indicated dilutions (solid bars) or cells were preincubated with DIPs for 24 h before virus was added (checkered bars). Infectivity was measured at 72 h post infection. The Log transformed average of five independent experiments is shown. Error bars indicate SEM. (**B**) The experiment was carried out as described for panel A but Calu-3 cells were used as targets. The log transformed average of three independent experiments is shown. Error bars indicate SEM. Two tailed paired student T-test was used to assess statistical significance. p < 0.5 = *, p < 0.05 = **, ns: not significant.

Addition of DIPs at 24 h prior to virus resulted in increased antiviral activity in both MDCK and Calu-3 cells as compared to co-inoculation of cells with virus and DIP (Fig. 5). Further, S1 DIP showed increased antiviral activity as compared to S3 DIP in keeping with the increased inhibitory activity of S1 relative to S3 DI RNA in the minireplicon assay. Finally, the antiviral activity of S1S3 DIPs was comparable to that of the S1 DIP (Fig. 5), demonstrating that under the conditions chosen the presence of two DI RNAs instead of one did not augment antiviral activity.

## Discussion

DIPs are naturally occurring byproducts of IAV replication. They contain DI RNAs that inhibit IAV infection and could be developed for antiviral intervention^11,13,20^. However, it is unclear whether the presence of two DI RNAs within DIPs can augment antiviral activity as compared to otherwise isogenic counterparts. Here, we demonstrate that cell lines expressing PB2 and PA allow the generation of DIPs harboring segment 1- and 3-derived DI RNAs in the absence of WT virus. Moreover, we provide evidence that the presence of two DI RNAs does not enhance interferon induction and antiviral activity.

In our current study we chose to produce IAV DIPs with deletions in segments 1 and 3, since DI RNAs preferentially arise from segments 1, 2 and 3^16,17^. Further, we chose DI RNAs with medium sized internal deletions that result in readily detectable but not maximal antiviral activity (not shown). This approach was used to ensure that a possible potentiation of DIP antiviral activity due to the presence of two DI RNAs was readily detectable. In addition, it allowed us to attain DIP titers suitable for experimentation. Thus, increasing the deletion size does not only increase antiviral activity but also reduces the efficiency of DIP production (not shown), most likely due to DIP auto-inhibition^21^ and this problem was circumvented using the above described strategy.

DIPs may interfere with IAV infection in two ways. They are robust activators of the IFN system and potent inducers of ISGs, and the antiviral activity of ISG products is believed to contribute to DIP antiviral activity^22^. Moreover, the smaller size of DIP RNAs allows them to replicate faster than the corresponding WT DI RNAs, hence allowing DIPs to outcompete WT virus for cellular resources that limit genome replication^13^. We would have expected that the presence of two DI RNAs might increase sensing of DIP by RIG1 and MDA5 and/or might augment the DIP-induced limitation of resources for genome replication, which both should result in increased antiviral activity^23^. However, the presence of two DI RNAs did augment neither inhibition of genome replication nor induction of MxA or antiviral activity.

At present, we can only speculate why the presence of two DI RNAs did not increase antiviral activity. One potential explanation could be the above-mentioned auto-inhibition. The rescue of S1S3 DIPs yielded 100-fold lower titers as compared to S1 or S3 DIPs, in keeping with potentially increased self-inhibiting capacity of S1S3 DIPs relative to S1 or S3 DIPs. Thus, it is conceivable that auto-inhibitory activity might not only have limited S1S3 amplification in the complementary cell lines used for DIP production, but might also have limited S1S3 DIP amplification in IAV co-infected cultures, thereby limiting DIP antiviral activity. Meng and colleagues have demonstrated that S1-derived DI RNAs may inhibit the replication of S1, S2, and S3 segments^24^. Hence, it is possible that S3 DI RNA replication is limited by the presence of S1 DI RNA in cells inoculated with S1S3 DIP. Finally, it is noteworthy that our findings are consistent with work from Zhao and colleagues who showed that simultaneous expression of S1-, S2- and S3-derived DI RNAs did not enhance antiviral activity relative to the single DI RNAs both in cell culture and in animals^25^.

In conclusion, our study provides evidence that the presence of two DI RNAs in IAV DIPs does not enhance antiviral activity. This finding should contribute to current efforts to develop DIPs for antiviral activity and might promote our understanding of the role of DIP in IAV spread and pathogenesis.

## Materials and methods

### Plasmids and oligonucleotides

Sequences of PB1 and PA were optimized for efficient human and influenza A virus codon usage and for maximal divergence from original sequences to reduce the potential for recombination with WT viral sequences^26^. Optimized sequences were synthesized (GeneArt, Germany) and subcloned via NotI and XhoI into pQCXIP-mcs^27^. For insertion of alternative selections markers, we used a derivative vector, pQCXIP-Cherry-mcs, where a puromycin resistance-mCherry fusion gene had been inserted into pQCXIP-mcs using an EcoRV site in the vector and introducing a MunI site upstream of the puromycin resistance gene. The puromycin resistance-mCherry fusion was then replaced by the neomycin (from pcDNA3), hygromycin (from pGL4.32) and blasticidin (pcDNA6/TR) resistance genes, to give pQCXIN-mcs, pQCXIHy-mcs and pQCXIBL-mcs, respectively. Using these vectors, the retroviral plasmids pQCXIN-PB2opt, pQCXIBL-PAopt and pQCXIHy-PB1opt were generated by subcloning the respective genes via NotI and XhoI.

Plasmids to rescue influenza virus strains PR8wt^26,28^ and WSN^28^ have been published. To generate segment 1 (S1) derived DIPs with intermediate size regions encompassing the packaging regions (1032 bp, 356 bp 5’, 550 bp 3’), we used splice overlap PCR using primers fluA AarI-PB2-1G (5- CGATCACCTGCTCGAGGGAGCGAAAGCAGGTC)/ DIP-448-rev (5- CCCACTGTATTGGCCTCTTAAGCGGCCGCTGCGGTACCGGATCCCCGACGTATTTTGACTTG) and DIP-448-for (5- CAAGTCAAAATACGTCGGGGATCCGGTACCGCAGCGGCCGCTTAAGAGGCCAATACAGTGGG)/fluA AarI-PB2-2341R (5- CGATCACCTGCTCTCTATTAGTAGAAACAAGGTCGTTT). The generation of segment 3 (S3) derived DIPs with intermediate size regions encompassing the packaging regions of the respective segments (958 bp, 447 bp 5’, 484 bp 3’), also employed splice-overlap PCR using primers fluA AarI-PA1-1 (5- CGATCACCTGCTCGAGGGAGCAAAAGCAGGTAC-3)/ DIPS3-447mcs-rev (5- CATCTCCATTCCCCATTTTTAAGCGGCCGCTGCGGTACCAGATCTCTCAGATTTAATTTTATT-3) and DIPS3-447mcs-for (5-AATAAAATTAAATCTGAGAGATCTGGTACCGCAGCGGCCGCTTAAAAATGGGGAATGGAGATG-3)/fluA AarI-PA1-2233R (5- CGATCACCTGCTCTCTATTAGTAGAAACAAGGTACTT-3). Both assembled fragments were cloned into pHW2000GGAar by Golden Gate cloning as described earlier^29^. Sequences for S1 and S3 DI RNAs are provided in the Supplemental Fig. 2). Plasmids for expression of PB1, PB2, PA and NP^15^ were generated by PCR amplification of the coding sequences from IAV strain lvPR8^30^ and cloning into pCAGGS^31^.

### Cells and viruses

All cells were cultured at 37 °C and 5% CO_2_. Madin-Darby Canine Kidney cells (MDCK) were obtained from the U. Reichl (MPI for Dynamics of Complex Technical Systems, Magdeburg)^15^ and incubated in Glasgow Minimum Essential Medium (GMEM; Gibco) with 10% fetal bovine serum (FBS; PAN Biotech), penicillin (100 IU/ml) and streptomycin (100 μg/ml) (pen/strep; PAN Biotech). MDCK PB2/PA cells were cultured in the presence of 1.5 µg/ml puromycin and 500 µg/ml neomycin respectively. 293 T cells were obtained from the DSMZ, Braunschweig, and maintained in Dulbecco’s Modified Eagle Medium (DMEM; Gibco) containing 10% FBS and pen/strep (ACC 635). 293T PB2/PB1/PA cells were grown in the presence of 1.5 µg/ml puromycin, 500 µg/ml neomycin and 5 µg/ml blasticidin. These cells were engineered to coexpress PB1 in order to allow production of DIPs harboring up to three DI RNAs within future studies. Calu-3 cells were maintained in Minimum Essential Medium (MEM) with 10% FBS, pen/strep, 1 × non-essential amino acid solution (10 × stock, PAA) and 10 mM sodium pyruvate (Thermo Fisher Scientific). The identity of 293T and Calu-3 cells was confirmed by STR typing^32^. Species specificity of MDCK cells was confirmed by cytB sequencing^33^. Identity of all cell lines was also confirmed by morphological assessment. Finally, cell lines were regularly screened for mycoplasma contamination.

Influenza A virus A/WSN/33 adapted to spread in A549 cells was obtained from the strain repository of the IVM Muenster and used to assess antiviral activity of DIPs^34^. We also used a recombinant vesicular stomatitis virus (VSV) that expresses a dual reporter consisting of eGFP and firefly luciferase from an additional transcription unit located between the open-reading frames for the viral glycoprotein and polymerase^35^.

### Production of retroviral vectors

For the production of MLV particles, 293 T cells were seeded in T25 flasks at a concentration of 2 × 10^5^ cells/ml in DMEM. The next day, cells were transfected using the calcium phosphate transfection method with 6 µg of retroviral vector pQCXIP-PB2opt, pQCXIN-PB2opt, pQCXIBL-PAopt, or pQCXIHy-PB1opt along with 3 µg MLV-gag-pol plasmid and 3 µg VSV-G expression plasmid^36,37^. At 48 h post transfection, supernatant containing the MLV particles was harvested and cleared by passaging through a 0.45 µm filter and stored at − 80 °C for further use.

### Transduction and selection of cell lines

96 well plates were seeded with 5,000 (MDCK) or 10,000 (293 T) cells/well in 50 µl of cell culture medium. MLV transduction particles (100 µl per well) were added the next day followed by spinoculation at 4,000 × g for 30 min to enhance transduction efficiency. At 48 h post transduction, transduced cells were detached and seeded in 24 well plates in culture media supplemented with antibiotics. Selection was continued until untransduced control cells had died.

To generate double transduced cells, transductions were performed in sequential order by first transducing PB2 gene followed by selection. Expression of PB2 was confirmed by western blot. Subsequently, PB2 expressing cells were transduced for further expression of PB1 and PA proteins. For selection of stable cell lines, 1 µg/ml and 1.5 µg/ml of puromycin was used for 293T cells and MDCK cells respectively, and 500 µg/ml neomycin, and 5 µg/ml blasticidin were used for both 293 T cells and MDCKs.

### Immunoblot

MDCK and 293T cells stably expressing the IAV polymerase proteins were seeded in 6 well plates at a cell density of 2 × 10^5^ cells/well. The next day, cells were harvested, lysed in 200 µL of Laemmli SDS-PAGE sample buffer (5% glycerin, 1% SDS, 2.5% ß-mercaptoethanol, 0.5% Bromophenol blue, 0.5 mM EDTA, 0.5 M Tris pH 6.8) and heated at 95 °C for 10 min. For each sample, 10 µL were loaded on 12.5% polyacrylamide gels and separated via SDS-PAGE. Proteins were blotted onto a nitrocellulose membrane (GE health care) using a Mini-PROTEAN Tetra Cell (BioRad). Membranes were blocked by incubation in 5% skim milk diluted in PBS-0.1% Tween (PBS-T) for 1 h. Subsequently, membranes were incubated with primary antibodies against PB2 (1:1,000, GeneTex, Irvine, USA), PB1 (1:1,000, GeneTex) or PA (1:500, GeneTex). For analysis of the expression of ß-actin, membranes were cut at 50 kDa prior to staining with ß-actin antibody (1:1,000, Sigma-Aldrich). Incubation with primary antibodies was done overnight at 4 °C. The next day, membranes were washed three times in PBS-T and incubated with anti-rabbit HRP (horseradish peroxidase)-conjugated secondary antibodies (1:10,000, Dianova) for one-hour at room temperature, followed by additional washing steps in PBS-T. In order to visualize protein bands, chemiluminescent substrate HRP juice plus (PJK) was added to the membranes and signals recorded with a ChemoCam imager (Intas).

### Minireplicon assay

293T cells were seeded in 12 well plates at a concentration of 2 × 10^5^ cells/well. Cells were transfected following an established protocol^15^ Briefly, cells were cotransfected with pCAGGS plasmids encoding PB1 (10 ng), PB2 (10 ng), PA (10 ng), NP (100 ng), reporter segment encoding firefly luciferase (50 ng) and plasmid encoding a DI RNA or an empty plasmid (concentrations indicated in figures). Cells were washed 6 h post transfection and fresh DMEM was added. After 24 h post transfection, cells were harvested and firefly luciferase activity was measured by using a Beetle-Juice Luciferase substrate (PJK). The values were recorded on a Plate Chameleon V reader (Hidex) using Microwin 2000 software.

### Production of DIPs

T25 flasks were seeded with a coculture of 293T cells stably expressing PB1, PB2, PA (1.4 × 10^6^ cells) and MDCK cells stably expressing PB2 and PA (0.4 × 10^6^ cells) in DMEM growth medium (Gibco). For the production of DIPs encoding two DI segments derived from IAV genomic segments 1 and 3 (S1S3 DIPs), cells were cotransfected with plasmids encoding DI RNA derived from segments 1 and 3 of PR8 origin, jointly with plasmids encoding IAV genomic segments 2 and 4 to 8 of WSN origin using the calcium phosphate method. Similarly, for production of DIPs expressing single DI segments with a deletion in segment 1 (S1 DIPs) or segment 3 (S3 DIPs), cells were cotransfected with 7 IAV genomic segments and either one DI segment derived from S1 (for S1 DIPs) or one derived from S3 (for S3 DIPs). After overnight incubation, cells were washed once with PBS and fresh DMEM infection medium (2% FBS, 1% pen/strep, without trypsin) was added. As negative control, parental MDCK and 293T cells were also transfected. Supernatants were harvested at 3, 5 and 7 days post transfection, cleared by centrifugation at 1500 × g for 10 min and stored at − 80 °C for further use. Titers for DIP supernatants were determined by focus formation assay on PB2, PA expressing MDCK cells, as described^15^.

### Characterization of DIP integrity

Integrity of DIPs was controlled by segment-specific RT-PCR. For the isolation of viral RNA from DIPs, 1.5 mL of DIP supernatant were centrifuged at 13,300 rpm for 2 h and total viral RNA from the resulting pellet was extracted using the RNeasy Mini kit (Qiagen) following the manufacturer’s instructions. 0.1 µg RNA was used as template for cDNA synthesis using the SuperScript III First-Strand Synthesis System (ThermoFisher Scientific). cDNAs corresponding to S1, S3 or S1S3 DIPs was amplified using segment specific PCR employing published primers^38^ fluA PB2-1 (5-AGCRAAAGCAGGTCAATTATATTCA)/ fluA PB2-2341R (5-AGTAGAAACAAGGTCGTTTTTAAACTA) for S1 and fluA PA-1 (5- AGCRAAAGCAGGTACTGATYCGAAATG)/ fluA PA-2233R (5- AGTAGAAACAAGGTACTTTTTTGGACA) for S3.

### Antiviral activity of DIPs

In order to assess antiviral activity of DIPs, MDCK cells were seeded at a concentration of 10,000 cells/well in 96 well plates and co-infected with A/WSN/33 (MOI 0.1) or VSV (MOI 0.01) jointly with DIPs (MOI 1 and tenfold dilutions) for one hour in GMEM growth medium. Alternatively, cells were incubated with the DIPs 24 h prior to infection with A/WSN/33 or VSV. Co-infected cells were washed one hour post coinfection and GMEM growth medium was added. Supernatants were harvested 72 h post coinfection and infectious titer was quantified by focus formation assay on MDCK cells, as described^39^. For analysis of DIP antiviral activity in Calu-3 cells, cells were seeded at 20,000 cells/well in 96 well plates in MEM growth medium and either co-infected with A/WSN/33 (MOI 0.001) and DIPs (MOI 0.1 and tenfold dilutions) or preincubated with DIPs 24 h prior to infection with A/WSN/33. After one hour, co-exposed cells were washed and MEM growth medium was added. Supernatants were harvested 72 h post infection and infectious titer was quantified by focus formation assay on MDCK cells.

### Quantitative real time PCR analysis

The induction of MxA by S1, S3 and S1S3 DIPs was assessed by qRT PCR. For this, Calu-3 cells were seeded in 12 well plates at 4 × 10^5^ cells/well and inoculated with A/WSN/33 (MOI 0.1), DIPs (S1, S3 or S1S3 MOI 0.1) or pan-IFNα (100 U/ml, PBL Assay Science) in MEM. As negative controls, cells were treated with fresh MEM or control supernatants obtained by using parental MDCK and 293T cells for DIP rescue. Following inoculation, cells were incubated for one hour, then washed once with PBS, and cultured in fresh MEM. At 24 h post treatment, cells were harvested and total RNA was extracted using the RNeasy Mini kit (Qiagen) following the manufacturer’s instructions. Extracted RNA was treated with RNase free DNase (New England BioLabs, NEB) and quantified using a NanoDrop spectrophotometer. For cDNA synthesis, 0.5 µg of RNA was used as template using the SuperScript III First-Strand Synthesis System (ThermoFisher Scientific), following the protocol for random hexamers in a total volume of 20 µl. Subsequently, 1 µl of cDNA was analyzed utilizing the QuantiTect SYBR Green PCR Kit (Qiagen). All samples were tested in triplicates on a Rotorgene Q device (Qiagen) for transcript levels of ß-actin (ACTB, internal transcript control) and myxovirus resistance protein A (MxA, interferon-stimulated gene) using published primers ^40^. Transcript levels were calculated from cycle thresholds (Ct values) and data are shown as fold change expression of MxA relative to ACTB. The differential fold expression of the target gene over the internal control was calculated using the Livak method^41^ as 2^-ΔΔCt^ where: ΔCt = average Ct (target gene/MxA) – average Ct ( reference gene/ACTB) and ΔΔCt = ΔCt (values for experimental conditions) –ΔCt (values for control conditions).

### Statistical testing

Students two tailed t-test was applied for statistical testing. p < 0.5 = *, p < 0.05 = **, p < 0.005 = ***, ns: not significant.

## Supplementary Information

Supplementary Information 1.

Supplementary Information 2.

## Data Availability

All data in this study are included in the manuscript.
