# Supplementary material for: Evidence that two instead of one defective interfering RNA in influenza A virus-derived defective interfering particles (DIPs) does not enhance antiviral activity
Source: Sci Rep. 2021 Oct 14;11:20477. doi: 10.1038/s41598-021-99691-1 (PMC8516915; doi:10.1038/s41598-021-99691-1)
Supplement: Supplementary file 1 — Supplementary Information 1. [file 41598_2021_99691_MOESM1_ESM.pptx]

## Slide 1
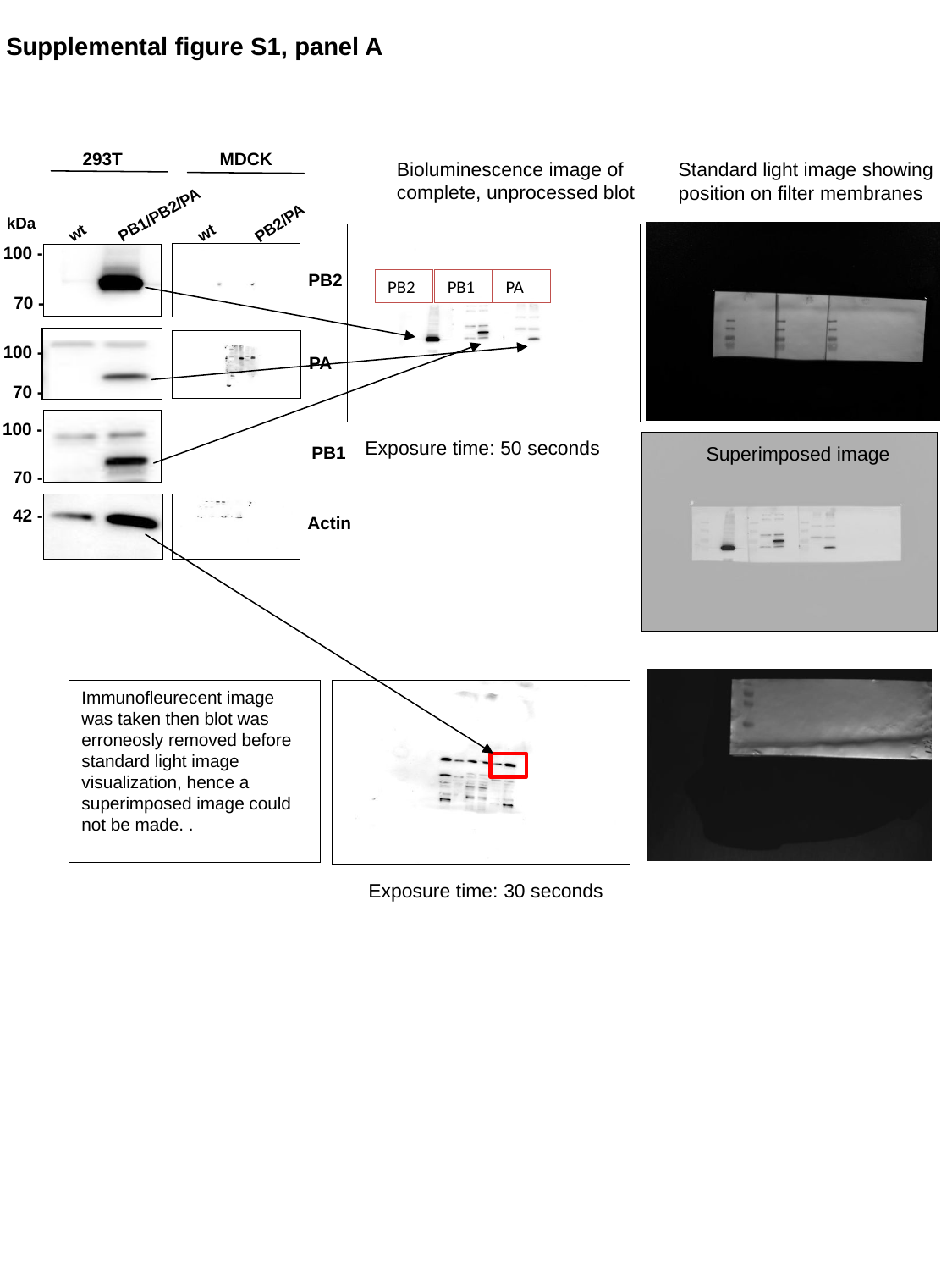

Supplemental figure S1, panel A
293T MDCK
PB1/PB2/PA
100 -
PB2
70 -
100 -
PA
70 -
Bioluminescence image of complete, unprocessed blot
Standard light image showing position on filter membranes
PB2/PA
kDa
wt
wt
PB2
PB1
PA
100 -
Exposure time: 50 seconds
PB1
Superimposed image
70 -
42 -
Actin
Immunofleurecent image was taken then blot was erroneosly removed before standard light image visualization, hence a superimposed image could not be made. .
Exposure time: 30 seconds

## Slide 2
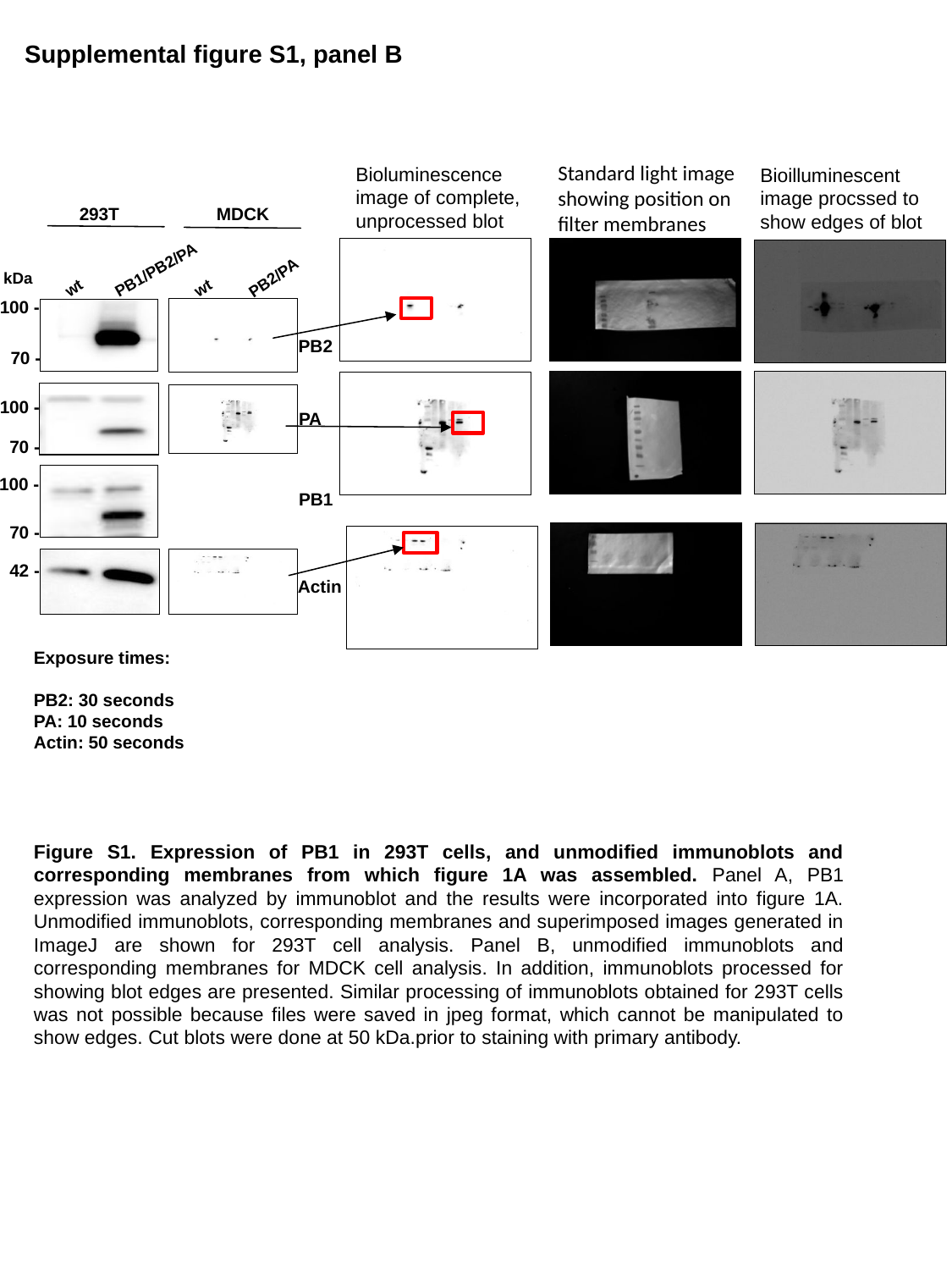

Supplemental figure S1, panel B
Standard light image showing position on filter membranes
Bioluminescence image of complete, unprocessed blot
Bioilluminescent image procssed to show edges of blot
Exposure times:
PB2: 30 seconds
PA: 10 seconds
Actin: 50 seconds
293T MDCK
PB1/PB2/PA
100 -
PB2
70 -
100 -
PA
70 -
PB2/PA
kDa
wt
wt
100 -
PB1
70 -
42 -
Actin
Figure S1. Expression of PB1 in 293T cells, and unmodified immunoblots and corresponding membranes from which figure 1A was assembled. Panel A, PB1 expression was analyzed by immunoblot and the results were incorporated into figure 1A. Unmodified immunoblots, corresponding membranes and superimposed images generated in ImageJ are shown for 293T cell analysis. Panel B, unmodified immunoblots and corresponding membranes for MDCK cell analysis. In addition, immunoblots processed for showing blot edges are presented. Similar processing of immunoblots obtained for 293T cells was not possible because files were saved in jpeg format, which cannot be manipulated to show edges. Cut blots were done at 50 kDa.prior to staining with primary antibody.
