# Supplementary material for: Evidence that two instead of one defective interfering RNA in influenza A virus-derived defective interfering particles (DIPs) does not enhance antiviral activity
Source: Sci Rep. 2021 Oct 14;11:20477. doi: 10.1038/s41598-021-99691-1 (PMC8516915; doi:10.1038/s41598-021-99691-1)
Supplement: Supplementary file 2 — Supplementary Information 2. [file 41598_2021_99691_MOESM2_ESM.docx]

**Supplemental figure S2: Sequences of S1 and S3 DI RNA**

**S1 DI RNA**

**1 AGCGAAAGCA GGTCAATTAT ATTCAATATG GAAAGAATAA AAGAACTACG AAATCTAATG**

**61 TCGCAGTCTC GCACCCGCGA GATACTCACA AAAACCACCG TGGACCATAT GGCCATAATC**

**121 AAGAAGTACA CATCAGGAAG ACAGGAGAAG AACCCAGCAC TTAGGATGAA ATGGATGATG**

**181 GCAATGAAAT ATCCAATTAC AGCAGACAAG AGGATAACGG AAATGATTCC TGAGAGAAAT**

**241 GAGCAAGGAC AAACTTTATG GAGTAAAATG AATGATGCCG GATCAGACCG AGTGATGGTA**

**301 TCACCTCTGG CTGTGACATG GTGGAATAGG AATGGACCAA TAACAAATAC AGTTCATTAT**

**361 CCAAAAATCT ACAAAACTTA TTTTGAAAGA GTCGAAAGGC TAAAGCATGG AACCTTTGGC**

**421 CCTGTCCATT TTAGAAACCA AGTCAAAATA CGTCGGGGAT CCGGTACCGC AGCGGCCGCT**

**481 TAAGAGGCCA ATACAGTGGG TTTGTAAGAA CTCTGTTCCA ACAAATGAGG GATGTGCTTG**

**541 GGACATTTGA TACCGCACAG ATAATAAAAC TTCTTCCCTT CGCAGCCGCT CCACCAAAGC**

**601 AAAGTAGAAT GCAGTTCTCC TCATTTACTG TGAATGTGAG GGGATCAGGA ATGAGAATAC**

**661 TTGTAAGGGG CAATTCTCCT GTATTCAACT ATAACAAGGC CACGAAGAGA CTCACAGTTC**

**721 TCGGAAAGGA TGCTGGCACT TTAACTGAAG ACCCAGATGA AGGCACAGCT GGAGTGGAGT**

**781 CCGCTGTTCT GAGGGGATTC CTCATTCTGG GCAAAGAAGA CAAGAGATAT GGGCCAGCAC**

**841 TAAGCATCAA TGAACTGAGC AACCTTGCGA AAGGAGAGAA GGCTAATGTG CTAATTGGGC**

**901 AAGGAGACGT GGTGTTGGTA ATGAAACGGA AACGGGACTC TAGCATACTT ACTGACAGCC**

**961 AGACAGCGAC CAAAAGAATT CGGATGGCCA TCAATTAGTG TCGAATAGTT TAAAAACGAC**

**1021 CTTGTTTCTA CT**

**S3 DI RNA**

**1 AGCAAAAGCA GGTACTGATC CAAAATGGAA GATTTTGTGC GACAATGCTT CAATCCGATG**

**61 ATTGTCGAGC TTGCGGAAAA AACAATGAAA GAGTATGGGG AGGACCTGAA AATCGAAACA**

**121 AACAAATTTG CAGCAATATG CACTCACTTG GAAGTATGCT TCATGTATTC AGATTTTCAC**

**181 TTCATCAATG AGCAAGGCGA GTCAATAATC GTAGAACTTG GTGATCCAAA TGCACTTTTG**

**241 AAGCACAGAT TTGAAATAAT CGAGGGAAGA GATCGCACAA TGGCCTGGAC AGTAGTAAAC**

**301 AGTATTTGCA ACACTACAGG GGCTGAGAAA CCAAAGTTTC TACCAGATTT GTATGATTAC**

**361 AAGGAGAATA GATTCATCGA AATTGGAGTA ACAAGGAGAG AAGTTCACAT ATACTATCTG**

**421 GAAAAGGCCA ATAAAATTAA ATCTGAGAGA TCTGGTACCG CAGCGGCCGC TTAAAAATGG**

**481 GGAATGGAGA TGAGGCGTTG CCTCCTCCAG TCACTTCAAC AAATTGAGAG TATGATTGAA**

**541 GCTGAGTCCT CTGTCAAAGA GAAAGACATG ACCAAAGAGT TCTTTGAGAA CAAATCAGAA**

**601 ACATGGCCCA TTGGAGAGTC CCCCAAAGGA GTGGAGGAAA GTTCCATTGG GAAGGTCTGC**

**661 AGGACTTTAT TAGCAAAGTC GGTATTCAAC AGCTTGTATG CATCTCCACA ACTAGAAGGA**

**721 TTTTCAGCTG AATCAAGAAA ACTGCTTCTT ATCGTTCAGG CTCTTAGGGA CAACCTGGAA**

**781 CCTGGGACCT TTGATCTTGG GGGGCTATAT GAAGCAATTG AGGAGTGCCT GATTAATGAT**

**841 CCCTGGGTTT TGCTTAATGC TTCTTGGTTC AACTCCTTCC TTACACATGC ATTGAGTTAG**

**901 TTGTGGCAGT GCTACTATTT GCTATCCATA CTGTCCAAAA AAGTACCTTG TTTCTACT**
